# Patient-Patient Similarity-Based Screening of a Clinical Data Warehouse to Support Ciliopathy Diagnosis

**DOI:** 10.3389/fphar.2022.786710

**Published:** 2022-03-25

**Authors:** Xiaoyi Chen, Carole Faviez, Marc Vincent, Luis Briseño-Roa, Hassan Faour, Jean-Philippe Annereau, Stanislas Lyonnet, Mohamad Zaidan, Sophie Saunier, Nicolas Garcelon, Anita Burgun

**Affiliations:** ^1^ Centre de Recherche des Cordeliers, INSERM, Sorbonne Université, Université de Paris, Paris, France; ^2^ HeKA, Inria, Paris, France; ^3^ Data Science Platform, Imagine Institute, Université de Paris, INSERM UMR 1163, Paris, France; ^4^ Medetia, Paris, France; ^5^ Service de Génétique, Hôpital Necker-Enfant Malades, Paris, France; ^6^ Service de Néphrologie, Hôpital Universitaire Bicêtre, Kremlin Bicêtre, France; ^7^ Laboratory of Renal Hereditary Diseases, Imagine Institute, Université de Paris, INSERM UMR 1163, Paris, France; ^8^ Department of Medical Informatics, Hôpital Necker-Enfant Malades, AP-HP, Paris, France

**Keywords:** patient similarity, electronic health records, medical concept embedding, rare disease diagnosis, screening, ciliopathy, unbalanced dataset

## Abstract

A timely diagnosis is a key challenge for many rare diseases. As an expanding group of rare and severe monogenic disorders with a broad spectrum of clinical manifestations, ciliopathies, notably renal ciliopathies, suffer from important underdiagnosis issues. Our objective is to develop an approach for screening large-scale clinical data warehouses and detecting patients with similar clinical manifestations to those from diagnosed ciliopathy patients. We expect that the top-ranked similar patients will benefit from genetic testing for an early diagnosis. The dependence and relatedness between phenotypes were taken into account in our similarity model through medical concept embedding. The relevance of each phenotype to each patient was also considered by adjusted aggregation of phenotype similarity into patient similarity. A ranking model based on the best-subtype-average similarity was proposed to address the phenotypic overlapping and heterogeneity of ciliopathies. Our results showed that using less than one-tenth of learning sources, our language and center specific embedding provided comparable or better performances than other existing medical concept embeddings. Combined with the best-subtype-average ranking model, our patient-patient similarity-based screening approach was demonstrated effective in two large scale unbalanced datasets containing approximately 10,000 and 60,000 controls with kidney manifestations in the clinical data warehouse (about 2 and 0.4% of prevalence, respectively). Our approach will offer the opportunity to identify candidate patients who could go through genetic testing for ciliopathy. Earlier diagnosis, before irreversible end-stage kidney disease, will enable these patients to benefit from appropriate follow-up and novel treatments that could alleviate kidney dysfunction.

## 1 Introduction

Rare disease patients that have delayed diagnosis present disease progression, incorrect treatment, and complications that may be irreversible. For example, a significant proportion of patients having ciliopathies are diagnosed when they have kidney failure and about half of patients on kidney transplantation waitlists are classified as undetermined diagnosis (Schrezenmeier et al., 2021).

Ciliopathies are an expanding group of rare and severe genetic diseases related to the abnormal structure and function of cilia, ubiquitous cellular organelles involved in controlling key signaling pathways during development and tissue homeostasis. Cilia dysfunction can lead to diseases with a broad spectrum of clinical manifestations ranging from embryo fetal lethality and individual organ malformation, to multisystemic defects. Until now, there have been no treatment for ciliopathies, but only maintenance and palliative care. Meanwhile several candidate drugs are currently tested in animal models or in *in vitro* models (Stokman et al., 2021).

Two major obstacles must be overcome to provide early diagnosis to ciliopathic patients: the phenotypic and genetic heterogeneity of patients, and the growing pace at which new clinical phenotypes are being described. Nowadays, more than 50 clinically and genetically overlapping ciliopathy disorders linked to variants in about 180 established ciliopathy-associated genes have been reported (Reiter and Leroux, 2017). Despite this progress, our knowledge of ciliopathies is far from complete, as new clinical phenotypes are still being described, shedding new light on the role of the primary cilium in health and disease (Shamseldin et al., 2020).

A conspicuous example of the complexity behind the diagnosis of ciliopathies is the recent discovery of ciliary genes pathogenic variants in end-stage kidney disease adult cohorts: the *NPHP1* homozygous locus-deletion was found in up to 0.9% frequency in adults raging 18–50 years of age (Snoek et al., 2018), along with a 0.3% frequency of other known ciliopathy-related gene mutations (Groopman et al., 2019). These studies, together with the fact that nonspecific clinical presentation is often missed due to a lack of suspicion for genetic tests, strongly suggest that ciliopathic patients are probably underdiagnosed.

In such complex situations, patient-patient similarity measures may be useful to search for potential ciliopathy patients in clinical data warehouses. Due to the wide adoption of electronic health records (EHR) systems in hospitals, patients’ data collected during care can be reused and mined to support diagnosis. To do so, we need a similarity model that considers the semantic relations between medical concepts and the different levels of relevance presented in patients’ EHRs—including incompleteness, inaccurate phenotyping, noisy phenotypes related to multiple comorbidities, and medical histories.

Recently, we developed a similarity method combining natural language processing (NLP) techniques, namely word embedding, and statistical modeling, to demonstrate the feasibility of screening a small patient cohort of 79 ciliopathies and 200 controls (Chen et al., 2021). The results showed a significant improvement in the enrichment of the number of ciliopathy patients among the top-ranked patients, compared with the baseline method that did not consider phenotype dependence and relevance.

The work presented here expands our previous preliminary study, as we 1) further assessed the adequacy of other existing embeddings for modeling medical concept dependence, 2) leveraged the similarity model by considering each diagnosis of ciliopathy as index (as opposed to using average similarity with all diagnosed patients) to take into account the high heterogeneity of ciliopathies, and 3) applied the developed model to two large-scale unbalanced datasets containing approximately 10,000 and 60,000 controls with kidney manifestations in the clinical data warehouse.

## 2 Material and Methods

### 2.1 Clinical Data Warehouse and Patient Phenotyping

This study was conducted as part of the C’IL-LICO project. This project was approved by the French National Ethics and Scientific Committee for Research, Studies and Evaluations in the field of Health (CESREES) under the number #2201437. It aims to develop transformative diagnostic, prognostic, and therapeutic approaches for patients suffering from ciliopathies. As a national reference center for rare and undiagnosed diseases, the Necker Children’s Hospital hosts the Imagine Research Institute, whose data repository contains more than 1800 patients with proven or suspected ciliopathy disorders. More than 1100 of them have bi-allelic variants in one causative gene identified. The clinical data warehouse (Dr. Warehouse) of Necker/Imagine contains EHR data from more than 700,000 patients. The high throughput phenotyping module within Dr. Warehouse (Garcelon et al., 2018) is based on the extraction of phenotypes encoded with the Unified Medical Language System (UMLS), a large thesaurus of medical terms and concepts from more than 200 different vocabularies. We used a definition of “phenotype” based on the UMLS, i.e., any concept assigned to 1 of the 12 semantic types (UMLS 2019AB release) belonging to the “Disorder” Semantic Group (McCray et al., 2001).

### 2.2 Patient Selection and Study Design

We considered two groups of patients: patients with ciliopathy disorders and non-ciliopathy controls.

Based on Dr. Warehouse, 329 patients with proven or suspected ciliopathy disorders had been followed at least once at Necker Children’s Hospital with EHR data available. To ensure inclusion of only patients with sufficient EHR information to characterize their health condition, we focused on patients with at least four distinct UMLS phenotype concepts. The concepts corresponding to the diagnosis of a ciliopathy, such as “*Nephronophthisis*” (*C0687120*), were removed to avoid bias.

The patient similarity method was applied to screen patients in Dr. Warehouse that had at least one kidney manifestation, to identify potential undiagnosed ciliopathy patients. More precisely, the target population for screening was selected as patients who had any automatically extracted UMLS phenotype concept subsumed by the term *“Kidney Diseases” (C0022658)* excluding known ciliopathies. This cohort is referred to as “other-nephropathy” controls. As the control cohort was built automatically based on the UMLS phenotype extraction of any kidney-related signs (from mild signs such as “polyuria” to end-stage kidney disease), it included patients of all ages (pediatrics and adults) and all types (native or transplant kidney disease).

### 2.3 Embeddings for Concept Similarity

The semantic similarity between two concepts was calculated using the cosine similarity between two embeddings. The embeddings of UMLS concept unique identifiers (CUIs) derived from a collection of 2.5 million French clinical narratives from Dr. Warehouse provided a good performance with the similarity method in a previous feasibility study (Chen et al., 2021). This embedding is referred to as cui_embd_fr in the following sections. We further assessed the adequacy of other existing embeddings for modeling medical concept dependence, including cui2vec and HPO2Vec+. cui2vec (Beam et al., 2019) contains embeddings of 109,053 UMLS CUIs, derived from multiple sources of medical data, including an insurance claims database of 60 million patients, 1.7 million full-text biomedical journal articles, and 20 million clinical notes. HPO2Vec+ (Shen et al., 2019) contains embeddings of 7258 terms from the Human Phenotype Ontology (HPO) trained from biomedical knowledge resources, such as Online Mendelian Inheritance in Man (OMIM), Orphanet, for HPO terms. To compare with the other two UMLS CUI embeddings (cui_embd_fr and cui2vec), the mapping between HPO terms and UMLS terms provided by the HPO consortium (HPO format-version: 1.2; data-version: releases/2020–12-07) was considered. Embedding of each HPO term was associated with all corresponding UMLS terms.

### 2.4 Patient-Patient Similarity

The similarity between two patients was calculated using an adjusted average best-match method, i.e., for each concept of each patient, the best match concept from the other patient was identified as the one that maximized the concept similarity, then concept similarities for all pairs of concepts were weighted averaged according to the relevance of each concept to each patient. More details are provided in (Chen et al., 2021).

Based on the pairwise patient similarity, we would like to measure an overall similarity between a patient and a group of patients, i.e., the diagnosed ciliopathy cases, to estimate the probability that the patient has ciliopathy. The first idea would be to consider the average similarity to all diagnosed cases. Intuitively, the closer patients are to the centroid of all diagnosed cases, the more likely they are to belong to the ciliopathy group. However, the underlying hypothesis is that all diagnosed cases are similar to each other and form a homogeneous group, which is inappropriate for ciliopathy because of its high clinical heterogeneity. Another option is to consider the maximum similarity to all diagnosed cases, i.e., the closer patients are to any of the diagnosed cases, the more likely they are to have ciliopathy. However, as we use EHR data that may contain relevant information but also noises, it may bring high uncertainty (analogy to “overfitting” in machine learning). We thus considered the average similarity to the five most similar diagnosed ciliopathy cases (referred to as max5-average in the following sections) to improve the robustness. This process shares the idea with a k-nearest neighbor classification where the neighbors are searched within the set of cases and an average similarity to searched neighbors was considered for ranking. The average, max, and max5-average correspond, respectively, to the average similarity to “all case neighbor,” “1-nearest case neighbor” and “5-nearest case neighbor.” Then all patients from the target screening population were ranked in the order of decreasing “overall” similarity (average, max, or max5-average).

We also considered the average similarity to each subtype of ciliopathies (such as nephronophthisis, Senior-Loken syndrome, Jeune syndrome, etc.) to measure the overall similarity between a patient and a subgroup of diagnosed ciliopathy cases. The final rank was based on the smallest rank obtained from all subtype averages (referred to as best-subtype-average in the following). An illustrative example is given in Table 1.

**TABLE 1:** Illustrative example best-subtype-average ranking model.

	Subtype 1		Subtype 2		Subtype 3		Smallest rank	Final rank
	Avg. Similarity	Rank	Avg. Similarity	Rank	Avg. Similarity	Rank		
Patient 1	0.9	1	0.5	1	0.1	3	1	1
Patient 2	0.8	2	0.2	3	0.7	1	1	1
Patient 3	0.7	3	0.4	2	0.6	2	2	3

### 2.5 Evaluation Measures

The proposed approach aims at screening for likely undiagnosed ciliopathy cases in a specific population. To evaluate the performance, EHR data from diagnosed ciliopathy cases and “other-nephropathy” controls were pooled. Each diagnosed case was considered as an index. The patient-patient similarity was calculated between each patient (case or control) and each index. The self-similarity (similarity between a patient and themselves) was set to NA. Then all patients were ranked with different ranking models (average, max, max5-average, and best-subtype-average) as described previously.

Based on these ranking models, the first k top-ranked patients were predicted as suspected ciliopathy patients for some fixed k. Then the most common measures of evaluation, such as precision and recall, can be determined at k. More precisely, the precision (or positive predictive value) is calculated as the proportion of true ciliopathy cases at top k to the total number of patients predicted to be ciliopathy cases (k). The recall (or sensitivity) is the proportion of true ciliopathy cases at k to the total number of true cases. Evaluation of the models was based on the precision-recall curves and partial receiver operator characteristic (ROC) curves. As the “full” curves are not very informative for the screening task with a large number of negative conditions, we focused on the top-ranked list. The minimum number of patients that would need to be screened to detect m true ciliopathy cases for m = 5, 10, 50, 100 were reported.

## 3 Results

### 3.1 Patients and Phenotyping

The ciliopathy data set comprises 253 previously diagnosed ciliopathy cases with at least four distinct UMLS phenotypes automatically extracted from their EHR data in Dr. Warehouse.

Two control cohorts were considered. First, we reused the set of “other-nephropathy” patients in Dr. Warehouse presented in our previous study (Chen et al., 2019) as the control cohort 1, which comprised 10,462 patients with the same inclusion criteria based on the number of minimum distinct UMLS phenotypes. Only “active” patients with the latest record after January 1, 2017, in Dr. Warehouse were included in this set. All patients (253 cases and 10,462 controls) presented 8698 distinct UMLS phenotypes. Based on this dataset, we evaluated different embeddings and different ranking models.

**FIGURE 1:**
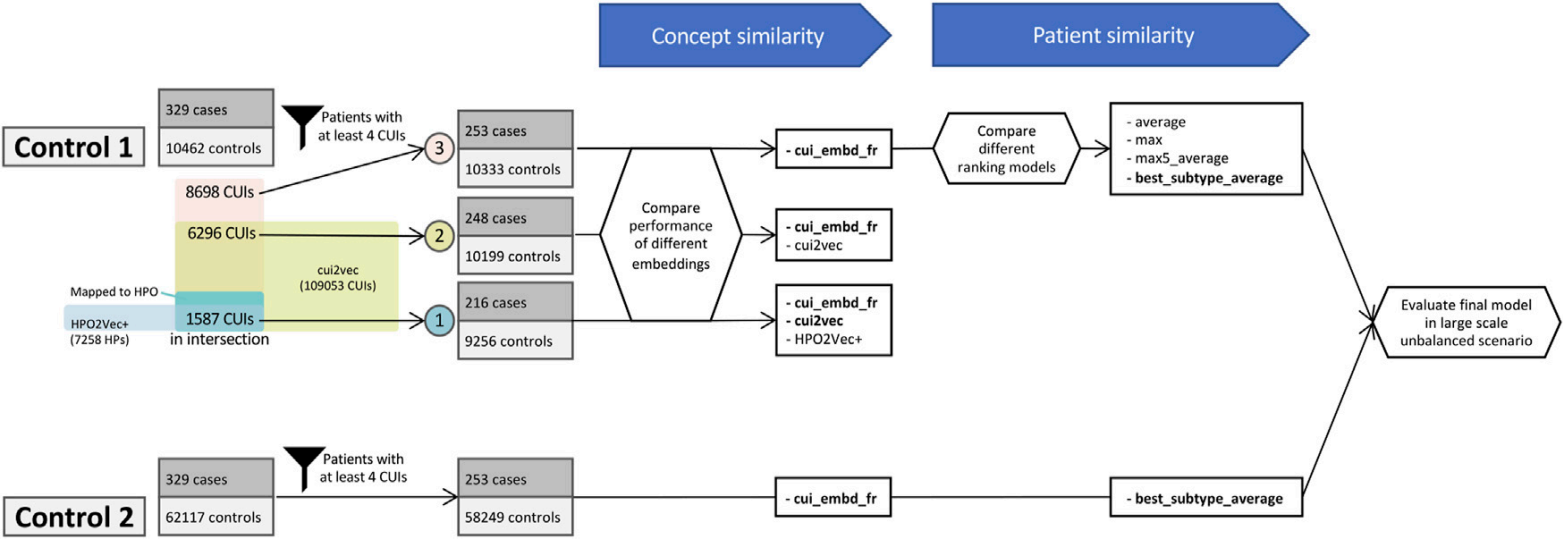
Overarching flow diagram.

Then, to assess the performance of our proposed method on a larger scale dataset with a more imbalanced case-control ratio, we built a second control cohort with the same method but removed the restriction on the date of the latest follow-up in Dr. Warehouse, which thus includes all very old and inactive patients as well. The second control cohort initially comprised 62,117 “other-nephropathy” patients, and after applying the same exclusion criteria based on the minimum number of phenotypes, 58,249 patients were retained. In this case, all patients (253 cases and 58,249 controls) presented 12,128 distinct UMLS phenotypes. An overarching flow diagram is shown in Figure 1.

### 3.2 Comparison of Different Embeddings

#### 3.2.1 Coverage of Different Embeddings

Using control cohort 1, cui_embd_fr was calculated for all 8698 UMLS phenotypes. As for cui2vec, since UMLS is a large thesaurus containing more than 4 million CUIs, although the pre-trained cui2vec contains 109,053 CUIs, only 72.4% of extracted UMLS phenotypes (6296 of 8698) were available in the pre-trained cui2vec. The 10 terms that were most frequently absent were *“Pyothorax-Associated Lymphoma” (C1709781)*, “*Transplant*” *(C3841811)*, “*Monoclonal*” *(C0746619)*, “*Organ finding*” *(C0941132)*, “*Urine microscopy leukocytes present finding*” *(C0555120)*, “*Ring dermoid of cornea*” *(C1867155)*, “*Immunosuppression*” *(C4048329)*, “*Anticoagulation* (*finding*)” *(C2919015)*, “*Therapy cessation*” *(C1699848*), and “*Peroxisome Biogenesis Disorder*, *Complementation Group R*” (*C1866352*). Among them, three absent terms correspond to noisy extraction, mainly due to French ambiguous abbreviations, such as “*Pyothorax-Associated Lymphoma*” - “*pal*”, “*Ring dermoid of cornea*” - “*rdc*”, and “*Peroxisome Biogenesis Disorder*, *Complementation Group R*” - “*cgr*”.

Regarding HPO2Vec+, only 24.5% of extracted UMLS terms were successfully mapped to HPO terms (2134 of 8698) via the conversion algorithm provided by HPO. The 10 most frequent unmapped terms were “*Systemic arterial pressure*” *(C1272641)*, “*Hypertrophy*” *(C0020564)*, “*Recurrence (disease attribute)*” *(C2825055)*, “*Hypersensitivity*” *(C0020517)*, “*Cyst*” *(C0010709)*, “*Communicable Diseases*” *(C0009450)*, “*Cicatrix*” *(C2004491)*, “*Urate level - finding*” *(C0729829)*, “*Disease regression*” *(C0684320)*, and “*Androgen-Insensitivity Syndrome*” *(C0039585)*. Among them, some terms do not exist in HPO as they are not considered as phenotypes in HPO, such as “*Systemic arterial pressure*”; and most terms could not be mapped due to lack of precision, such as “*Cyst*” in UMLS vs. “*Renal cyst*”/“*Pulmonary cyst*”/“*Bone cyst*”/etc., in HPO. Among 2134 mapped HPO terms, 1587 were available in HPO2Vec+. The 5 most frequent absent HPO terms were *“Moderate albuminuria” (HP:0012594)*, “*Crackles*” (*HP:0030830*), “*Macroscopic hematuria*” *(HP:0012587)*, “*Renal tubular epithelial necrosis*” (*HP:0008682*), and “*Addictive behavior*” (*HP:0030858*). The mutual coverages of the different embeddings are shown in Figure 1.

#### 3.2.2 Performance of Different Embeddings

With the aim of assessing the adequacy of different embeddings for modeling medical concept dependence, we first restricted this analysis to the UMLS concepts available in both pre-trained cui2vec and HPO2Vec+. The inclusion criteria based on the minimum number of phenotypes was applied based on this list of 1587 phenotypes, i.e., each patient presenting at least four of these concepts. The dataset comprised 9472 patients (216 ciliopathy patients and 9256 controls). The number of phenotypes for each ciliopathy patient ranged from 4 to 130, with a median value of 18 (interquartile 8–33). The number of phenotypes for each control patient ranged from 4 to 161, with a median value of 18 (interquartile 9–34). There is no significant difference between ciliopathy cases and controls regarding the number of phenotypes.

**FIGURE 2:**
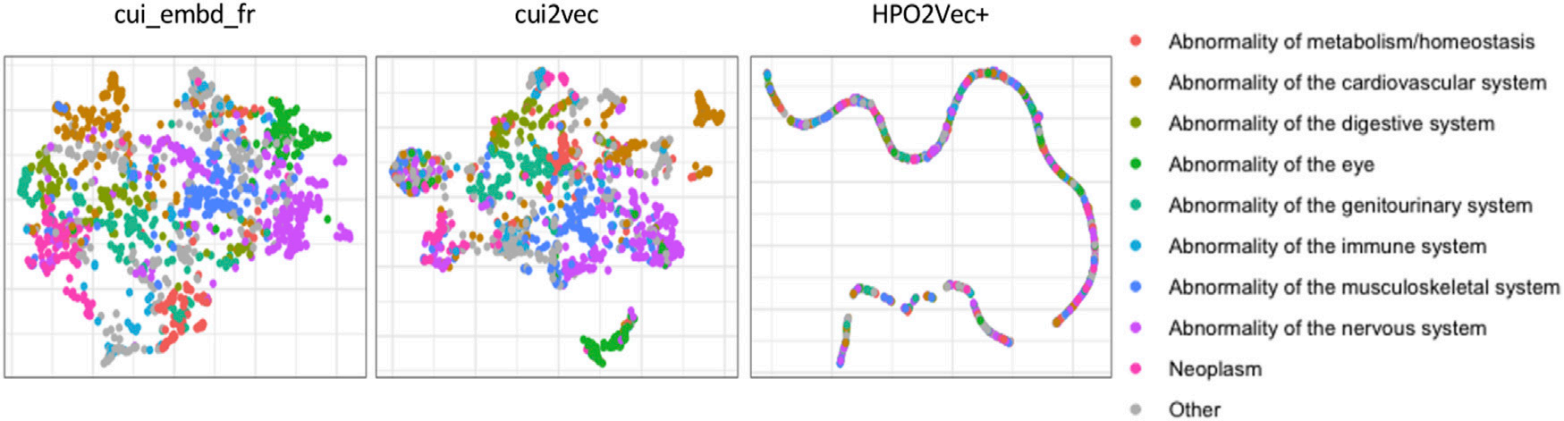
Phenotype projection of different medical concept embeddings. The phenotypes were colored according to their hierarchical categories in HPO.

We first projected the three embeddings of 1587 phenotypes onto a two-dimensional plot for visualization using Uniform Manifold Approximation and Projection (UMAP) (Figure 2). The phenotypes were colored according to their hierarchical categories in HPO. More precisely, we considered the direct descendants of “Phenotypic abnormality” in HPO, such as “Abnormality of the nervous system,” “Abnormality of the genitourinary system,” etc., as phenotype categories. As shown in Figure 2, phenotypes belonging to the same category were generally better grouped together with cui_embd_fr and cui2vec than with HPO2Vec+.

**FIGURE 3:**
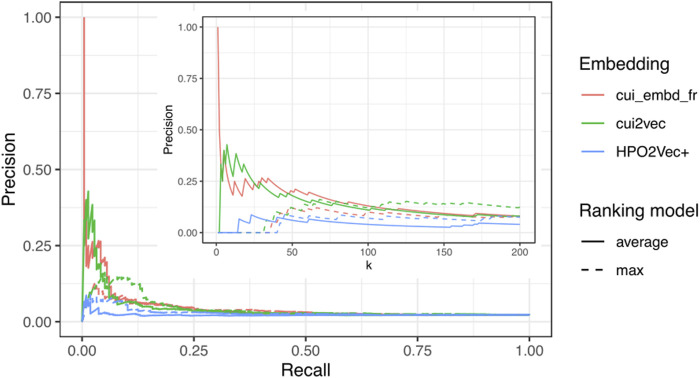
Precision-recall curves with a zoom for small values of k (k<=200) for different embeddings.

All three embeddings (cui_embd_fr, cui2vec, and HPO2Vec+) were then used to calculate the concept similarity for the same dataset. Patient-patient similarities were calculated with adjusted average best-match aggregation between all patients (cases and controls) and each ciliopathy case. All patients were ranked according to the average and max similarity to all ciliopathy cases. In terms of screening performance, the results are summarized in Table 2. The minimum numbers of patients that would need to be screened to detect m out of 216 ciliopathy cases are shown for different embeddings and different values of m. The precision-recall curves are shown in Figure 3 with a zoom for small values of k. The two UMLS CUI embeddings (cui_embd_fr and cui2vec) outperformed HPO2Vec+. Using less than one-tenth of learning sources, cui_embd_fr achieved comparable performances compared to cui2vec, which implied the interest in a center and language specific embedding.

**TABLE 2:** Comparison of different embeddings.

Evaluation set	Embedding	Ranking model	k for m true positives			
			m = 5	m = 10	m = 50	m = 100
1587 CUIs	**cui_embd_fr**	average	**19**	**49**	**1056**	3781
		max	47	99	1071	**3036**
	cui2vec	average	**13**	**63**	**1247**	3826
		max	48	67	1253	**3530**
	hpo2vec	average	155	355	2263	4765
		max	**61**	**136**	**1598**	**3908**
6294 CUIs	**cui_embd_fr**	average	**7**	**50**	957	3,211
		max	25	72	**697**	**2546**
	cui2vec	average	**13**	69	1201	3,160
		max	23	**51**	**711**	**2728**
8696 CUIs	**cui_embd_fr**	average	**15**	**43**	847	3,158
		max	18	80	**538**	**2175**

The best results were in bold.

As discussed before, a significant proportion of UMLS concepts did not have any corresponding terms in HPO, and some mapped HPO terms were not in the pre-trained HPO2Vec+, including concepts that may be important for characterizing ciliopathy and other nephropathy patients, such as *“Diabetic Nephropathy” (C0011881)* not mapped to HPO, and “*Moderate albuminuria*” *(C1654921, HP:0012594)* mapped to HPO but absent in the pre-trained HPO2Vec+. Therefore, we enlarged the dataset by including all UMLS phenotypes with the two CUI embeddings available and further compared cui_embd_fr and cui2vec. This dataset comprised 10,447 patients (248 ciliopathy cases and 10,199 controls), representing in total 6294 distinct UMLS phenotypes. The results are shown in Table 2. Compared to the first dataset with only 1578 phenotypes, the performances with both CUI embeddings were improved, as more patient information was considered. The two CUI embeddings still provided comparable results, confirming the interest of a language and center specific embedding since cui_embd_fr was derived from much smaller learning sources than cui2vec.

Finally, as a supplementary analysis, we included all 8696 UMLS phenotypes and re-calculated the performance of cui_embd_fr. The results were not further improved (Table 2), suggesting the presence of redundancies and noises in EHR data.

### 3.3 Comparison of Different Ranking Model

Based on the results shown in Section 3.2, we focused on cui_embd_fr in the following analysis to include all extracted UMLS phenotypes as it provided the same level of performance as cui2vec and much better performance than HPO2Vec+.

#### 3.3.1 Subtypes of Ciliopathies

**FIGURE 4:**
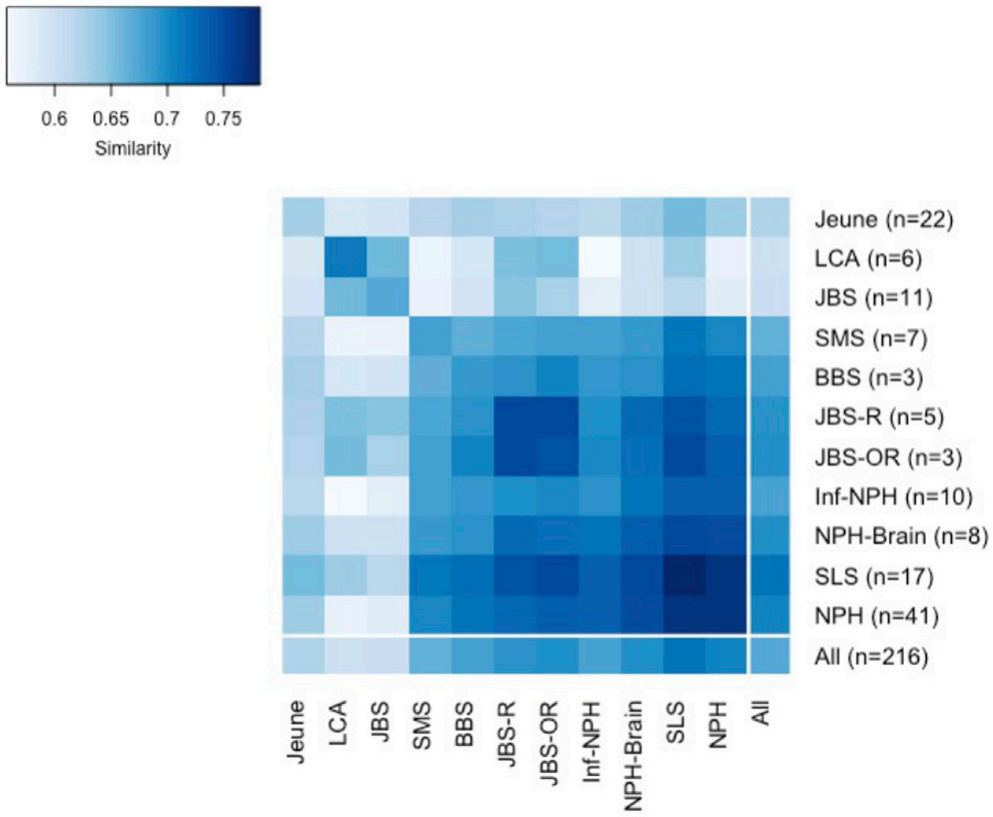
Internal-external group average similarities for 11 most frequent ciliopathy subtypes.

The genetic data for all ciliopathy patients with at least four distinct “Disorder” UMLS phenotypes were collected. The precise diagnosis was made and normalized to Orphanet if possible. Pathogenic variants of 39 ciliopathy-related genes were identified in 169 patients (66.8%). Eleven ciliopathy types were present in at least three patients in our data set: Nephronophthisis (NPH), Jeune syndrome (Jeune), Senior-Loken syndrome (SLS), Joubert syndrome (JBS), infantile nephronophthisis (Inf-NPH), nephronophthisis with brain developmental damages (NPH-Brain), Saldino-Mainzer syndrome (SMS), Leber congenital amaurosis (LCA), Joubert syndrome with renal defect (JBS-R), Bardet-Biedl syndrome (BBS), and Joubert syndrome with oculorenal defect (JBS-OR), with 3–41 patients. The most frequent was nephronophthisis. Other diagnoses with only one or two patients were not considered for calculating subtype-average similarity. The internal-external group average similarities for these 11 most frequent ciliopathy subtypes are shown in Figure 4, illustrating the high heterogeneity of ciliopathies and the overlap of phenotypic representation among the different subtypes.

#### 3.3.2 Comparing Performance Between Ranking Models

**FIGURE 5:**
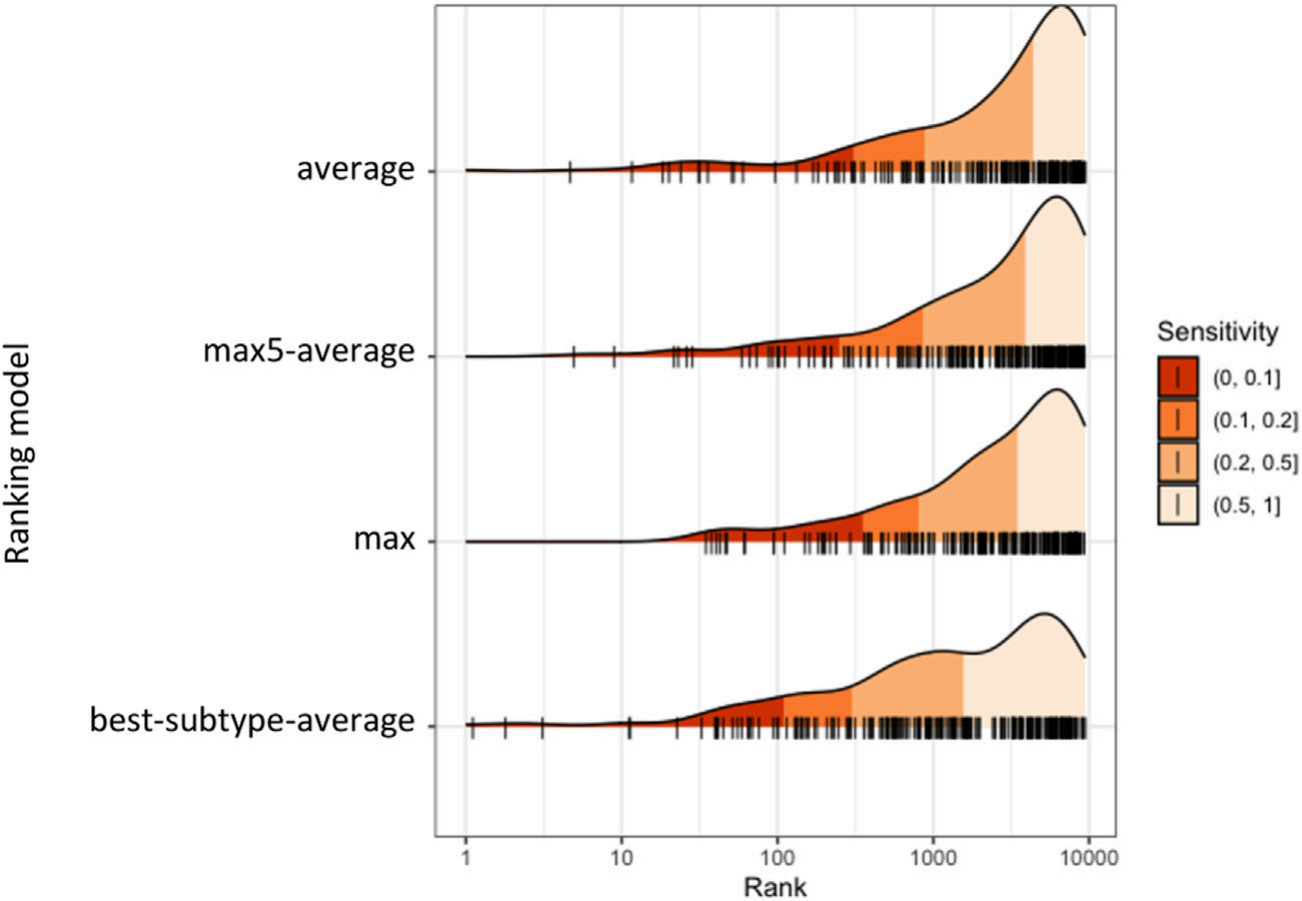
Distribution of ranks of diagnosed ciliopathy cases obtained from different ranking models.

For each patient (ciliopathy case or control), the average similarities with each considered subtype were calculated, and the ranks were obtained for each subtype as rank_NPH, rank_Jeune, rank_SLS, etc. The minimum value of all ranks was used for the final ranking. We compared the performance between the four ranking models: mean, max, max5-average, and best-subtype-average. The distribution of the four ranks is shown in Figure 5. The 10, 20, and 50% quantile were indicated, which represent the minimum number of patients that would need to be screened to include x% of the true ciliopathy cases, thus correspond to the true positive rate (TPR, or sensitivity, or recall) of 10, 20, and 50%. The minimum number of patients that would need to be screened to detect 50 out of 248 ciliopathy cases (corresponding to a TPR of 20.2%) was 272 for best-subtype-average (precision of 18.4%), compared to 957, 697, and 614 for average, max, and max5-average ranking models (precision of 5.2, 7.2, and 8.1%, respectively), which indicated that the ranking model with best-subtype-average performed the best for true cases.

**FIGURE 6:**
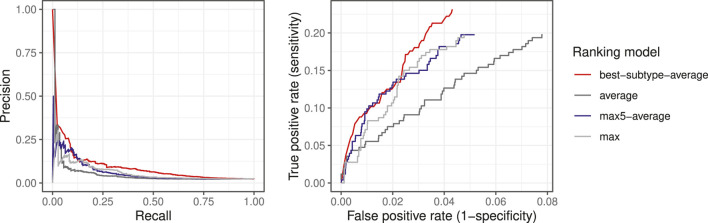
Precision-recall curves and the partial ROC curves for different ranking models.

The precision-recall curves and the partial ROC curves are shown in Figure 6 for the four ranking models. We observed that the ranking model by best-subtype-average can significantly improve the performance, especially for small values of k, which is of particular interest as we aim to identify suspected ciliopathy patients at the top-ranked list who would benefit from genetic testing. Given that random testing applied to the same data set would require testing 2106 patients in order to detect 50 ciliopathy patients (prevalence in the data set of 248/10,447), we can conclude that the best-subtype-average ranking model with medical concept embedding improved detection of ciliopathy patients by more than sevenfold.

### 3.4 Performance on Larger Scale Dataset With Extremely Unbalanced Case-Control Ratio

We developed this method to screen large clinical data warehouses to detect undiagnosed patients with rare conditions. In such situations, the absolute value of evaluation metrics may be meaningless as an unbalanced case-control ratio may inflate type I error. For example, in a dataset with 100 cases and 100 controls (1:1 case-control ratio), an algorithm with false positive rate (FPR, or probability of false alarm) of 5% can achieve 95% precision@100; while in an imbalanced dataset with 100 cases and 1000 controls (1:10 case-control ratio), the precision@100 decreases to 50% with the same FPR of 5%, and in an extremely imbalanced dataset with 100 cases and 10,000 controls (1:100 case-control ratio), the precision@100 may fall to nearly 0 with an algorithm with FPR under 5%. In a real-life application, a rare disease is defined in Europe as a disease affecting less than 1 in 2000 citizens, which is an extremely imbalanced situation. Therefore, we further assessed the performance of our proposed method on a larger dataset with diagnosed ciliopathy patients pooled with patients from control cohort 2, and compared the results between the two situations with a random model.

The proposed ranking model using cui_embd_fr and best-subtype-average similarity was applied, and the results are shown in Table 3. The prevalence of cases using control cohort 2 was about one-fifth of the prevalence using control cohort 1. Therefore, with a random model to detect the same number of true cases, the minimum number of patients that would need to be screened increases fivefold. For example, to detect 30 out of 253 ciliopathy patients by random testing, 1244 and 6937 patients would need to be screened based on a prevalence of cases using control cohort 1 and control cohort 2, respectively; while with our proposed method, 102 and 290 patients would have to be screened in the two case-control ratio settings, corresponding to, respectively, 12.2- and 23.9-fold improvement in the enrichment of ciliopathy patients among the top-ranked patients. The enrichment factor is not constant, implying the effectiveness of our method in a large-scale extremely unbalanced dataset.

**TABLE 3:** Performance in two unbalanced datasets.

	Control cohort 1			Control cohort 2		
Case-control ratio	253:10,235			253:58,249		
Prevalance	∼ 2%			∼ 0.4%		
k for m true positives	m = 50	m = 30	m = 20	m = 50	m = 30	m = 20
Random model	2072	1244	829	11561	6937	4625
Ranking model with cui_embd_fr and best-subtype-average similarity	272	102	62	939	290	130
Emrichment factor	7.6	12.2	13.4	12.3	23.9	35.6

### 3.5 Clinical Evaluation of Top Similar Control Patients

We performed a more detailed evaluation of the EHRs of the top-ranked patients. More precisely, we went through the final ranking of patients from most to least similar to the ciliopathies, and asked ciliopathy experts (SS, MZ) to review the EHR of the 20 first met controls to analyze their profiles. At this point, 17 ciliopathies were included as well.

Among 20 control patients, 14 patients were diagnosed with or suspected of a genetic disease. In 4 of them, the cause of the genetic disease was pathogenic variants related with ciliary function (2 confirmed cases, 2 suspected), such as *PMM2* (Dorval et al., 2021) and *HNF1B* (Gresh et al., 2004), carrying overlapped phenotypes with ciliopathies. Ten patients were diagnosed with or suspected to have a genetic disease that affects the process of development; they presented multisystemic malformations or developmental disorders overlapped with ciliopathies (4 confirmed, 6 suspected), such as congenital anomalies of the kidney and urinary tract (CAKUT), Kabuki syndrome, and Dravet syndrome, 9 out of 10 being differential diagnosis of ciliopathies. The 8 patients classified as suspected had not been provided with a molecular diagnosis yet.

Five patients were unlikely to have any genetic disorders, and the following diagnosis was established: Lithium induced nephropathy (1 case), IgA nephropathy (1 case), post-pneumococcal hemolytic-uremic syndrome (1 case), and non-genetic neonatal disorders (2 cases) (oligohydramnios and cortical necrosis). A high similarity with ciliopathies was due to comorbidities. Finally, 1 patient died before receiving a diagnosis.

Therefore, our results can be summarized as follows: if we consider the set of 37 similar patients, we obtain 17 ciliopathies and 4 cases of genetic disease caused by a pathogenic variant related with ciliary function (21 “true positives” (57%) if we define a true positive as having a mutation related with cilia), and 9 more patients with a differential diagnosis whose phenotypic descriptions overlap with ciliopathies (30 cases where the similarity algorithm was successful (81%)).

## 4 Discussion

Underdiagnosis and delayed diagnosis is a common key challenge for many rare diseases (Faviez et al., 2020). Several studies confirmed the central role of data mining techniques applied to EHR data to identify cases of rare conditions, either genetic or not. For example, Doyle et al. reported that lack of suspicion, nonspecific symptoms, and co-existing conditions are frequent diagnosis difficulties for nontuberculous mycobacterial rare lung disease, and screening for likely undiagnosed cases in the primary-care population is a feasible solution (Doyle et al., 2020). Savolainen et al. demonstrated the feasibility of using EHR data to identify undiagnosed patients suffering from Gaucher disease, a rare inherited multiorgan disorder that is often delayed diagnosed due to a broad spectrum of symptoms and lack of disease awareness (Savolainen et al., 2021). At Necker/Imagine Institute, Dr. Warehouse integrated with NLP of unstructured narrative reports was demonstrated valuable to make diagnosis of Dravet syndrome earlier (Barco et al., 2021), and to identify 2 undiagnosed patients with a *KCNA2* variant in neurodevelopmental syndrome (Hully et al., 2021) based on similarity matching with other patients from the local data warehouse.

The similarity-based approach that we have developed re-used a comprehensive phenotypic description of patients based on their EHR data to detect ciliopathy patients in a clinical data warehouse. Unlike other studies using only a limited set of features presented in EHR, such as International Classification of Disease (ICD) codes (Griffiths et al., 2020), or a set of pre-defined disease specific phenotypes (Savolainen et al., 2021), we extracted all UMLS medical concepts in EHRs. Our results showed that the performance can be improved by including more phenotypes (6296 vs. 1578 phenotypes). However, using all 8698 medical concepts extracted from EHRs did not lead to further improvement compared with 6296 phenotypes, suggesting the presence of redundancies and noises in EHR data. The dependence and relatedness between phenotypes were taken into account in our patient similarity model through medical concept embedding. We did not consider the information content (IC)-based semantic similarities, such as the Resnik (1995) and Lin (1998) similarity, or the pathway distance-based semantic similarities, such as the one mentioned in Yang et al. (2021), because these similarities require an ontological structure more formal than the UMLS Metathesaurus. Moreover, some concepts can be close in the ontology, but very different from a semiological point of view as they have different pathogenesis: this is the case, for example, between “Type 1 diabetes” and “Type 2 diabetes”, or “chronic renal insufficiency” and “acute renal insufficiency.” The relevance of each phenotype to each patient was also considered by adjusted aggregation of phenotype similarity into patient similarity. As most of the NLP efforts were focused on English texts, using multilingual reference terminology enables us to leverage and to evaluate existing embeddings learned from English medical data sources for French clinical narratives. To address the phenotypic overlapping and heterogeneity of ciliopathies, each subtype was considered individually to calculate the average similarity. Then, as suggested by ciliopathy experts (McConnachie et al., 2021), the different subtypes of ciliopathy were considered as a continuum of disorders for the diagnosis task, and the minimum value of all ranks with different subtypes was considered. Our final ranking model based on the best-subtype-average outperformed other ranking models, which supports and reinforces this idea. We demonstrated the effectiveness of our screening approach in an unbalanced condition, using two control cohorts with about 1:40 and 1:200 case-control ratio, respectively, which is more in line with real-life rare disease diagnosis and also an important issue in big healthcare data as mentioned in Wolford et al. (2018). Twenty top-ranked control patients that were similar to ciliopathies patients were reviewed. Most of them (70%) were diagnosed with or suspected of a genetic disease that clinically overlaps with ciliopathies, involving genes either having direct impact on ciliary function, or relating to a differential diagnosis of ciliopathies. Such good performance is very encouraging and we plan to apply our algorithm to external data warehouses.

This kind of screening approach should be distinguished from diagnosis supporting systems that were developed for clinicians that face a new patient and have all information on desk: these systems aim at reducing miss rate (prioritizing recall/sensitivity), and a false alarm (type I error) is less critical in the diagnosis scenario. In contrast, our screening approach is an automated system expected to address underdiagnosis issues of rare diseases in a large clinical data warehouse. In that situation, the system uses routine care data with patient information that can be less precise and less comprehensive, and the objective is to maximize the hit (prioritizing precision). In doing so, we expect that top-ranked patients will benefit from genetic testing, and thus can be diagnosed earlier before the development of irreversible lesions. Therefore, the purpose is to find patients that should be tested and followed-up by experts. Moreover, it could be possible that some top-ranked patients presenting similar clinical profiles but not carrying a known pathogenic variant in ciliary genes may benefit from the same treatment.

As for ciliopathies, it could be interesting to apply our approach to screen populations known to be associated with a considerable proportion of ciliary disease, such as retinal dystrophy patients, as 30% of patients with isolated retinitis pigmentosa (RP, a genetic disorder of the eyes affecting 1 in 4000 people) are ciliopathy patients. We should notice that diseases’ prevalence in our evaluation datasets may be different from that in the general population. As Necker Children’s Hospital is a reference center for genetic diseases, the local data warehouse includes a larger number of patients with ciliary disease (in particular, renal ciliopathies) compared to other centers. Indeed, in our dataset 86% of diagnosed ciliopathy cases have kidney impairment extracted from EHR. Meanwhile, it is particularly challenging to distinguish between patients with isolated nephronophthisis and many other nephropathies, as renal impairments generally present with nonspecific features in isolated nephronophthisis patients. Therefore, we considered two nephropathy cohorts as control cohorts to evaluate our approach, the first one with about 10,000 patients, and the second one with about 60,000 patients. Both control cohorts were built automatically based on the UMLS phenotype extraction of any kidney-related phenotypes (from mild signs such as “polyuria” to end-stage kidney disease), including patients of all ages and all types (native or after kidney transplantation). Therefore, the inclusion of demographic data such as age at chronic kidney disease onset, and exclusion of phenotypes occurring after dialysis or kidney transplantation are likely to improve performance of such an approach. A pre-exclusion of patients unlikely to have genetic disorders (such as those diagnosed with a lithium-induced nephropathy or IgA nephropathy who were found among the top-ranked similar controls) could be considered as well.

There are several limitations in this study. As our proposed approach is trying to match a patient’s phenotypes to a subgroup of diagnosed ciliopathies in Dr. Warehouse, the performance highly depends on the quality of patient’s phenotyping from EHR for both cases and controls. In this work, patient phenotypes were extracted from EHRs in Necker Children’s Hospital and were not inclusive of medical visits and treatments outside Necker Children’s Hospital, thus the phenotyping can be incomplete. On the other hand, false positive extractions of phenotypes were observed, many of them being related to polysemy and abbreviation as shown in Section 3.2.1. Further efforts are required to improve the quality of phenotype extraction. In this work, we did not take into account the longitudinality of phenotypes. A patient was represented as a “bag” of all their phenotypes, including early signs, symptoms during disease progression, irreversible damages, and post-treatment symptoms (such as post-transplantation phenotypes). It would be interesting to use only phenotypes before the diagnosis as indexes to search for similar patients and eventually determine at which stage the presented phenotypes enable early diagnosis of ciliopathy (hopefully, before the onset of chronic kidney disease). Moreover, the phenotypic similarity should take into account the longitudinality to be able to distinguish, for example, two patients with both kidney and eye abnormalities, one patient with early onset of kidney disorders and progressive eye abnormality in adulthood and the other patient with eye abnormalities in infancy then late onset of renal affection. To address all these issues, solutions for automatically extracting phenotype temporal relations and their chronological timeline should be considered in the future.

Our results suggest several clinical and methodological perspectives. The next step will be to perform the similarity-based screening in other hospitals. As Necker Hospital is a national reference center for rare and undiagnosed diseases, it is less probable to identify mis/underdiagnosed patients without any genetic investigation in its local data warehouse. Therefore, we could expect even better results in other hospitals. Regarding the methodology, an embedding derived from a larger number of rare disease data sources may potentially improve the modeling of the dependence between medical concepts. Generating synthetic patients could be considered to better represent each subtype of ciliopathies. It is foreseeable that further improvements may be achievable by integrating complementary information. For example, disease descriptions extracted from HPO, Orphanet, and OMIM can be used to exclude patients who are similar to ciliopathy but even more similar to other diseases to address the differential diagnosis issue. In addition to the phenotypic similarity, patients’ biological data can also be integrated to the similarity model. In the future, for the diagnosed ciliopathy patients, omics data and new knowledge on pathways should be integrated together with clinical data to regroup clinically similar ciliopathies that may benefit from the same molecule.

## Data Availability

The datasets presented in this article are not readily available because institutional officials expressed concern about the inability to guarantee anonymity. Aggregate data and datasets containing coarse-grained phenotypes are available upon request to the corresponding author. Requests to access the datasets should be directed to XC, xiaoyi.chen@inserm.fr.
